# Hyperglycemia, diabetes, and coronary microvascular dysfunction in INOCA

**DOI:** 10.3389/fendo.2025.1719777

**Published:** 2025-12-01

**Authors:** Pasquale Mone, Fahimeh Varzideh, Fabio Minicucci, Maria Luisa D’Onghia, Giada Sabatelli, Luigi Savino, Marco Savino, Gaetano Mottola, Urna Kansakar, Gaetano Santulli

**Affiliations:** 1 Department of Medicine and Health Sciences “Vincenzo Tiberio”, University of Molise, Campobasso, Italy; 2 Casa di Cura “Montevergine”, GVM Care and Research, Mercogliano (Avellino), Italy; 3 Division of Cardiology, Albert Einstein College of Medicine, New York, NY, United States; 4 ASL (Local Health Authority) Napoli, Naples, Italy; 5 International Translational Research and Medical Education (ITME) Consortium, Joint Academic Research Unit, Department of Advanced Biomedical Sciences, “Federico II” University, Naples, Italy

**Keywords:** acute coronary syndrome, coronary microvascular dysfunction, diabetes mellitus, endothelial dysfunction, INOCA, microvascular angina, MINOCA, stress hyperglycemia ratio

## Introduction

Ischemia with no obstructive coronary artery (INOCA) is a relatively frequent condition in patients admitted for coronary angiography for angina and/or positive stress test, increasing the risk of hospitalizations and adverse events (1, 2). Approximately 70% of subjects undergoing coronary angiography for angina have no coronary obstruction with signs of ischemia (3–5).

Previous observations have evidenced that older age, hypertension, smoking, dyslipidemia, obesity, female sex, and diabetes mellitus were potential determinants of adverse outcomes in nonobstructive coronary artery disease (CAD) (6, 7).

The exact mechanisms underlying the pathophysiology of INOCA have not been fully determined; however, there are currently two main hypotheses that seem to prevail: coronary microvascular dysfunction (CMD) with microvascular angina (MVA) and epicardial coronary artery spasm (8). In particular, stress hyperglycemia has been advocated as a marker of adverse events in patients with cardiovascular diseases (9, 10).

We believe that hyperglycemia and diabetes may be pivotal in the onset of CMD and, as such, we summarized the available literature accordingly.

### Coronary microvascular dysfunction

INOCA is commonly defined by the following criteria (1, 11):

- Symptoms and objective evidence of myocardial ischemia;
- Non-obstructive coronary artery stenosis defined as <50% diameter reduction and/or fractional flow reserve >0.80; and
- Impaired coronary microvascular function defined as impaired coronary flow reserve (CFR) (≤2.0), abnormal coronary microvascular resistance [i.e., index of microcirculatory resistance (IMR) ≥25], and coronary microvascular spasm (defined as reproduction of symptoms and ischemic ECG shifts but no epicardial spasm during acetylcholine testing).

**Figure 1:**
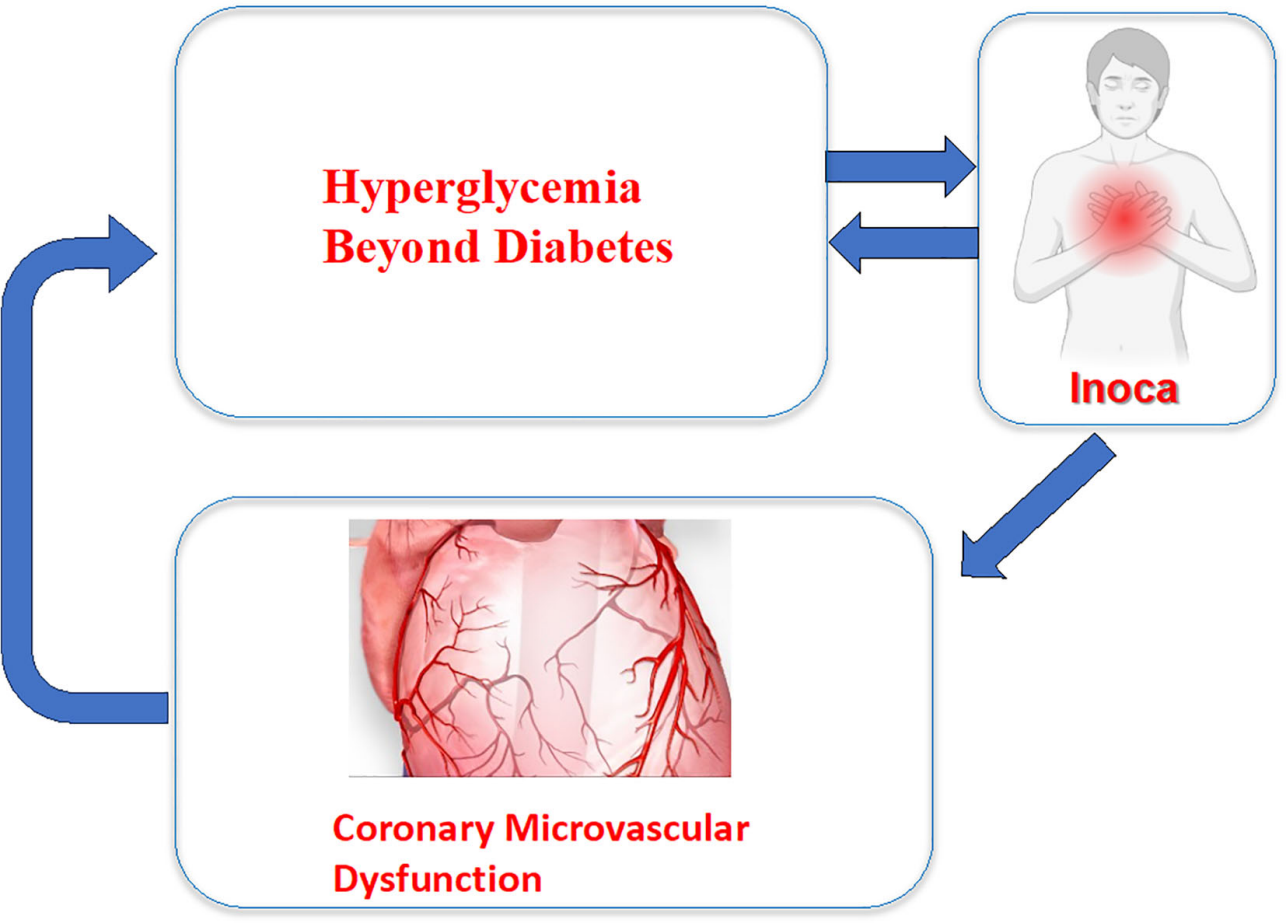
Coronary microvascular dysfunction in INOCA.

CMD is one of the main mechanisms advocated in INOCA (**Figure 1**). Comorbidities may drive an early CMD impairing microcirculation (12, 13). There are currently two endotypes of CMD leading to MVA: structural microcirculatory remodeling and functional arteriolar dysregulation (14–16).

A structural remodeling of the coronary microvasculature could decrease microcirculatory conductance and worsen oxygen delivery capacity (17). This condition is linked to the inward remodeling of coronary arterioles, with an increase in wall-to-lumen ratio, a loss of myocardial capillary density (capillary rarefaction), or both (18). Afterwards, there is a classic remodeling due to traditional comorbidities, atherosclerosis, left ventricular hypertrophy, or cardiomyopathies (7, 19, 20). As a result, there is a reduction of the vasodilatory range of the coronary microcirculation, limiting maximal blood and oxygen supply to the myocardium, as remodeled arterioles are hypersensitive to vasoconstricting stimuli (21). Finally, in response to adenosine, there are two characteristics of microcirculation:

1) a reduced CFR, and
2) an increase in minimal (hyperemic) microcirculatory resistance.

### Stress hyperglycemia and coronary artery disease

Stress hyperglycemia is a common condition in hospitalized patients and is defined as a transient hyperglycemia during illness due to inflammation, cortisol, and catecholamine activation (10, 22). Stress hyperglycemia is typical in CAD (23), and stress hyperglycemia ratio (SHR) is currently used as a marker in coronary patients (24). A recent investigation has demonstrated that elevated SHR is significantly associated with 1-year and long-term all-cause mortality, especially in subjects without diabetes (25). Furthermore, SHR was a significant predictor for adverse cardiovascular outcomes in patients with or without diabetes and three-vessel CAD (26). Consistent with the aforementioned study, a recent paper correlated SHR with the severity of CAD in both prediabetic and diabetic individuals (27). Intriguingly, SHR has been associated with CAD even in patients with acute coronary syndrome and may be a useful marker of risk stratification (28). A recent study highlighted an impact of SHR in myocardial infarction with no-obstructive coronary artery (MINOCA) (29). SHR has been linked to adverse events even in coronary artery bypass grafting (CABG) (30, 31) like in multivessel CAD based on brain natriuretic peptide levels (32). Finally, SHR could be a marker after percutaneous coronary intervention (33).

### Effects of diabetes on coronary microcirculation

Coronary microcirculation plays a seminal role in the regulation and homeostasis of myocardial perfusion, regulating blood flow, fulfilling myocardial metabolic needs, and managing peripheral vascular resistance (34). Diabetes impairs coronary microcirculatory function as insulin resistance drives acute vascular responses during postprandial hyperglycemia (35–37). The effect of insulin resistance on microvascular dysfunction may not be due to diabetes and/or hyperglycemia (38, 39). Moreover, diabetes negatively impacts endothelial function driving CMD (40, 41), with myocardial fibrosis and dysfunctional remodeling driving alterations in left ventricular filling until overt diastolic dysfunction (42–45). In this scenario, diabetic patients have an increased risk of cardiovascular events than nondiabetic patients (46). Intriguingly, there is an increased risk of heart failure due to “diabetic cardiomyopathy”, which may be due to coronary dysfunction (47–50). Indeed, subjects with diabetic cardiomyopathy often present significant major epicardial coronary disease associated with heart failure with preserved ejection fraction (HFpEF) (51–54).

### Potential effects of hyperglycemia in coronary microcirculation beyond diabetes

Hyperglycemia beyond diabetes is a well-accepted risk factor for adverse events in patients with cardiovascular diseases and cardiovascular risk factors affecting endothelial function (55–58). Our group has published a recent paper in which we evidenced the role of stress hyperglycemia in the onset of hospitalization for chest pain in patients with INOCA (59). Still, we showed a significant effect of metformin treatment in the reduction of hospitalizations in hyperglycemic patients with INOCA with a reduction of oxidative stress in human coronary artery smooth muscle cells and endothelial cells (60). These results should be carefully analyzed because stress hyperglycemia should be considered a consequence of myocardial ischemia probably generated by CMD (61–64).

## Discussion and future perspectives

We speculate that hyperglycemia beyond diabetes may be a marker of CMD in INOCA. Of note, several studies are necessary to confirm our hypothesis. However, the detrimental effects of hyperglycemia on endothelium are well known. In this scenario, hyperglycemic patients should be treated before the onset of diabetes to reduce cardiovascular risk and preserve endothelial and coronary microvascular function. Metformin is an old drug that could be pivotal in the treatment of hyperglycemic patients with INOCA. Of note, treating all the risk factors with statins, ACE inhibitors, and beta-blockers may be crucial in improving the quality of life in INOCA.
